# Electrochemical Properties of Poly(Anthraquinonyl Sulfide)/Graphene Sheets Composites as Electrode Materials for Electrochemical Capacitors

**DOI:** 10.3390/nano4030599

**Published:** 2014-07-30

**Authors:** Wonkyun Lee, Shinya Suzuki, Masaru Miyayama

**Affiliations:** 1 School of Engineering, The University of Tokyo, 7-3-1 Hongo, Bunkyo-ku, Tokyo 113-8656, Japan; E-Mails: sin@fmat.t.u-tokyo.ac.jp (S.S.); miyayama@fmat.t.u-tokyo.ac.jp (M.M.); 2 CREST, Japan Science and Technology Agency, 4-8-1 Honcho, Kawaguchi, Saitama 332-0012, Japan

**Keywords:** Poly(anthraquinonyl sulfide), graphene, composite, electrochemical capacitors

## Abstract

Poly(anthraquinonyl sulfide) (PAQS)/graphene sheets (GSs) composite was synthesized through *in situ* polymerization to evaluate its performance as an electrode material for electrochemical capacitors. PAQS was successfully synthesized in the presence of GSs with uniform distribution. PAQS/GSs showed a pair of reversible redox peaks at around 0 V (*vs.* Ag/AgCl). The specific capacitance of PAQS/GSs was 349 F·g^−1^ (86 mAh·g^−1^) at a current density of 500 mA·g^−1^, and a capacitance of 305 F·g^−1^ was maintained even at a high current density of 5000 mA·g^−1^. The *in situ* polymerization of PAQS with GSs facilitated their interaction and enabled faster charge transfer and redox reaction, resulting in enhanced electrode properties.

## 1. Introduction

The increased demand for clean, renewable energy has led to intense research efforts into energy storage systems. Electrochemical capacitors (ECs) are of particular interest due to their high power density and long cycle life [1,2,3,4]. In particular, ECs using aqueous instead of organic electrolyte have the additional advantage of high safety. ECs can store energy via two mechanisms: electrostatic charge separation at the electrode/electrolyte interface, or Faradaic redox reaction at the surface of the active electrode material [5,6]. Recent studies have focused on improving the limited energy density of ECs by exploring high-capacitance electrode materials such as transition-metal oxides [7,8,9] and conducting polymers [10,11,12].

Quinone-based materials have attracted considerable attention as electrode materials for energy storage devices such as lithium-ion batteries [13,14,15,16,17], redox flow batteries [18], polymer/air batteries [19] and electrochemical capacitors [20,21,22]. Although quinone-based materials can provide high capacitance through a two-electron redox reaction, degradation of performance due to the dissolution of quinone molecules into both aqueous and organic electrolytes is a significant obstacle to their use in energy storage devices [14,21,22]. Recently, quinone polymer electrodes such as poly(2,5-dihydroxy-1,4-benzoquinone-3,6-methylene) (PDBM) [23], poly(2,5-dihydroxy-1,4-benzoquinonyl sulfide) (PDBS) [24] and poly(anthraquinonyl sulfide) (PAQS) [13,25] exhibited enhanced cycling stability with relatively high capacitance in lithium-ion batteries. Among these, PAQS was synthesized by a simple method and showed high reversible capacitance of 185 mAh·g^−1^ with good cyclability [13]. In the present study, we focused on PAQS as an electrode material for electrochemical capacitors. To the best of our knowledge, there are no reports on PAQS electrodes using aqueous electrolytes.

Graphene sheets (GSs) are single-atom-thick carbon sheets with outstanding properties that include large specific surface area [26,27], high electrical conductivity [28] and excellent mechanical performance [29]. Previous attempts to produce GSs composites with redox-active polymers resulted in composites that showed good performance due to the enhanced electrical conductivity of the polymers in combination with graphene [30,31,32].

Here, we synthesized PAQS in the presence of dispersed GSs (PAQS/GSs) to investigate the influence of *in situ* polymerization of PAQS with GSs on the electrode properties for electrochemical capacitors. This *in situ* polymerization was a simple process because of the favorable π–π interactions between PAQS and GSs. The microstructure and electrode properties of PAQS/GSs were evaluated in order to understand the reaction activity of PAQS in aqueous electrolyte and the effect of *in situ* polymerization with GSs.

## 2. Results and Discussion

### 2.1. FT-IR Spectra of GSs, PAQS and PAQS/GSs

**Figure 1:**
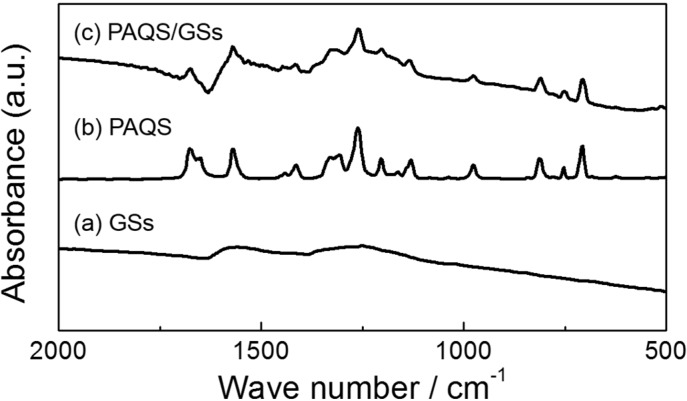
FT-IR spectra of (**a**) graphene sheets (GSs); (**b**) poly(anthraquinonyl sulfide) (PAQS); and (**c**) PAQS/GSs.

Figure 1 shows the FT-IR spectra of GSs, PAQS and PAQS/GSs. As shown in Figure 1a, the featureless FT-IR spectrum of GSs indicates that the peaks for the oxygen functional groups were almost completely removed by thermal exfoliation [33]. The FT-IR spectrum of PAQS shows absorbance peaks that are consistent with those of reported PAQS [13,25,34] (Figure 1b). The peaks at 1676 and 1568 cm^−1^ represent, respectively, C=O and C=C stretching vibrations of the anthraquinonyl group. The peaks at 1413 and 1129 cm^−1^ correspond to the stretching of the sulfur-disubstituted aromatic ring and the ring-sulfur, respectively. These results indicate that anthraquinone-based PAQS was successfully synthesized. As shown in Figure 1c, the absorbance peaks of PAQS/GSs were similar to those of PAQS, indicating that PAQS is well synthesized even in the presence of GSs.

### 2.2. Raman Spectra for GSs, PAQS and PAQS/GSs

**Figure 2:**
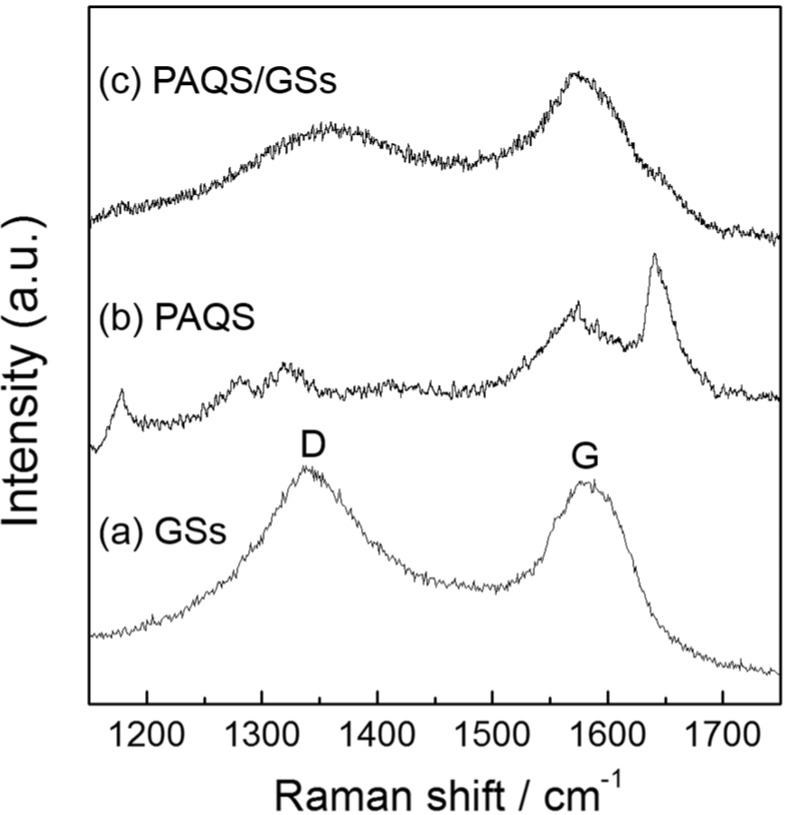
Raman spectra of (**a**) GSs; (**b**) PAQS; and (**c**) PAQS/GSs at an excitation wavelength of 514 nm.

Figure 2 shows the results of Raman spectra for GSs, PAQS and PAQS/GSs. In the GSs, two bands were observed at about 1340 and 1580 cm^−1^, corresponding to the G band and D band (Figure 2a). The G band is associated with the ordered sp2 carbon lattice and the D band originates from the structural defects and disorders in the graphene layer [35,36]. For PAQS, in-plane C–H bending vibrations and symmetric stretching of sulfur-disubstituted ring, and C=O stretching vibrations of the anthraquinonyl group appeared at 1177, 1570 and 1642 cm^−1^, respectively [37,38] (Figure 2b). For PAQS/GSs, two dominant peaks were observed similarly to GSs (Figure 2c). PAQS/GSs presents a shift in the G band towards lower wave numbers compared to GSs due to the π–π interactions between the polymer and graphene sheets, indicating that PAQS is well combined with the GSs [31,39]. The disappearance of in-plane C–H bending and C=O stretching vibrations of PAQS is another evidence of the π–π interactions [39]. The relatively weak Raman signal for PAQS is similar to that of highly dispersed polymers on graphene [40,41], which reveals that PAQS is well dispersed on the graphene sheets.

### 2.3. XRD Patterns for GSs, PAQS and PAQS/GSs

**Figure 3:**
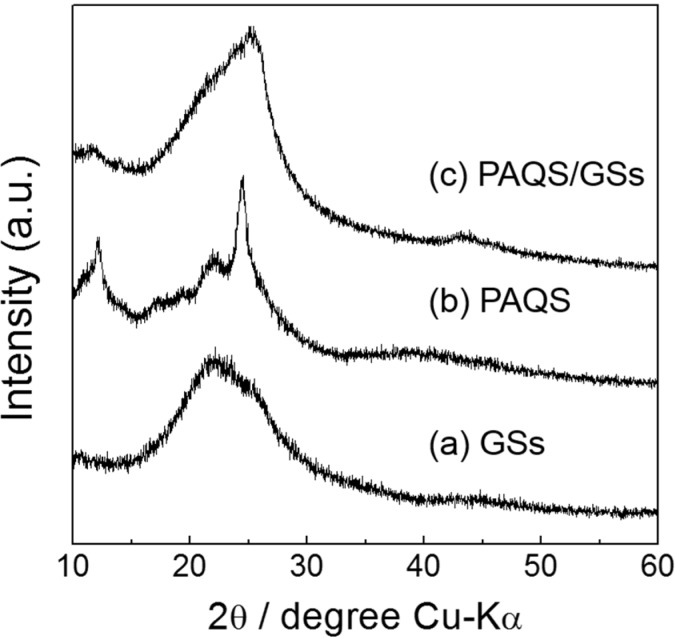
XRD patterns of (**a**) GSs; (**b**) PAQS and (**c**) PAQS/GSs.

Figure 3 shows the XRD patterns for GSs, PAQS and PAQS/GSs. As can be seen in Figure 3a, GSs exhibited a broad diffraction peak at around 2°–21°, indicating that graphene sheets are randomly stacked compared to graphite. For PAQS, the crystalline peaks appeared at 12°, 22° and 24°, which is consistent with previously reported data [13] (Figure 3b). The XRD patterns of PAQS/GSs exhibit one dominant diffraction peak similar to that of GSs but with the main peak position at 24°, indicating that the weak diffraction peak of PAQS is combined with that of GSs (Figure 3c). The results of FT-IR, Raman and XRD suggest that the synthesis of a homogeneous PAQS/GSs composite was successful.

### 2.4. N_2_ Adsorption-Desorption Isotherms of GSs, PAQS, PAQS/GSs(m) and PAQS/GSs

**Figure 4:**
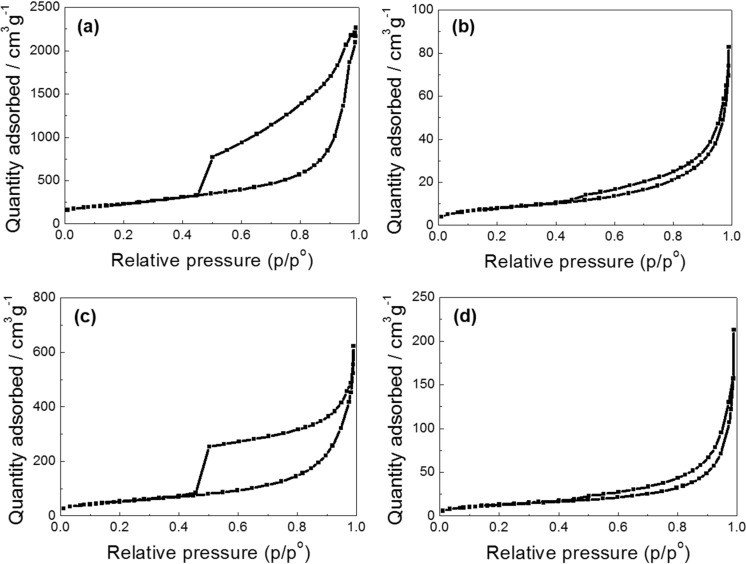
N_2_ adsorption-desorption isotherms for (**a**) GSs; (**b**) PAQS; (**c**) PAQS/GSs(m); and (**d**) PAQS/GSs.

To investigate the influence of *in situ* polymerization of PAQS with GSs on the electrode properties for electrochemical capacitors, we prepared and measured mechanically mixed PAQS/GSs(m) as a comparison material. Figure 4 shows the N_2_ adsorption-desorption isotherms of GSs, PAQS, PAQS/GSs(m) and PAQS/GSs. In the GSs, obvious hysteresis loop was observed at relative pressure ranging from 0.45 to 1.0, which is attributed to a mesoporous structure (Figure 4a). GSs exhibited a large BET surface area of 849 m^2^·g^−1^. As shown in Figure 4b, however, PAQS showed a BET surface area of 27 m^2^·g^−1^, which is attributed to the aggregation of polymers during the polymerization process. In the PAQS/GSs(m), a hysteresis loop which is originated from the structure of GSs was still observed, indicating that significant aggregation was not accompanied during mechanical mixing process (Figure 4c). PAQS/GSs(m) exhibited a BET surface area of 190 m^2^·g^−1^. As shown in Figure 4d, PAQS/GSs exhibited a decreased specific surface area of 42 m^2^·g^−1^ compared to GSs, and this is greatly attributed to the enhanced aggregation of the PAQS/GSs particles during the *in situ* polymerization.

### 2.5. Microstructures of GSs, PAQS, PAQS/GSs(m) and PAQS/GSs

**Figure 5:**
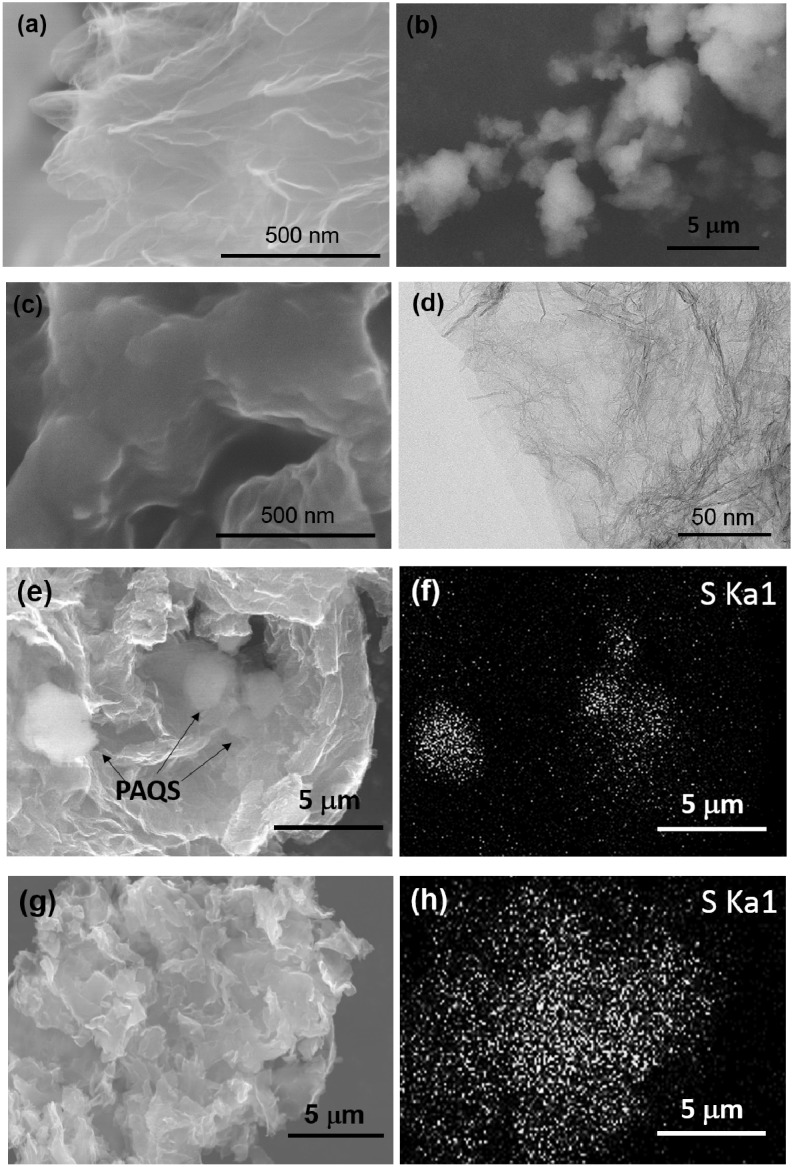
SEM images of (**a**) GSs; (**b**) PAQS; and (**c**) PAQS/GSs; (**d**) TEM image of PAQS/GSs. SEM images and energy-dispersive X-ray (EDX) sulfur elemental mapping images of (**e**,**f**) PAQS/GS(m) and (**g**,**h**) PAQS/GS.

Figure 5 illustrates the microstructures and morphology of GSs, PAQS, PAQS/GSs(m) and PAQS/GSs. As shown in Figure 5a, GSs has a wrinkled morphology, which is attributed to the insertion and removal of oxygen functional groups during the preparation process, and the graphene sheets were loosely agglomerated. The pure PAQS exhibits firmly aggregated structure with a size of 1–5 μm (Figure 5b). As shown in Figure 5c, there is no aggregation of PAQS particles in the PAQS/GS, and a smooth surface and increased thickness of sheets were observed. This reveals that the surface of GSs is covered with highly dispersed PAQS due to the favorable π–π interactions between PAQS and GSs [30]. A TEM image shows that the PAQS/GSs sheets are nearly transparent under electron irradiation, implying that PAQS is dispersed on graphene sheets with extremely thin morphology (Figure 5d). In the PAQS/GS(m), however, PAQS was not fully dispersed due to the recurrence of aggregation during the preparation process (Figure 5e). Energy-dispersive X-ray (EDX) spectroscopy elemental mapping was used to investigate the distribution of PAQS in the composite and mixture. The elemental mapping of sulfur on PAQS/GSs(m) shown in Figure 5f reveals the aggregated PAQS in the structure. In the case of PAQS/GSs, SEM images and elemental mapping of sulfur reveal that PAQS is uniformly dispersed on the GSs (Figure 5g,h).

### 2.6. Electrochemical Properties

#### 2.6.1. Cyclic Voltammograms

**Figure 6:**
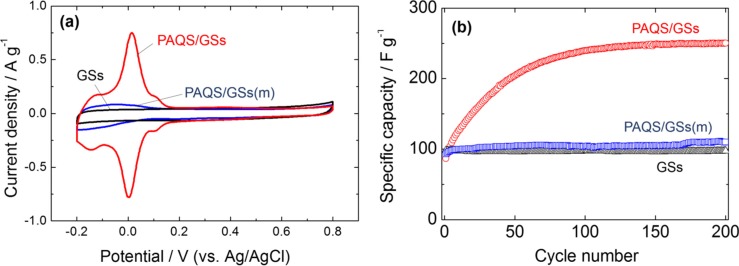
(**a**) Cyclic voltammograms at a scan rate of 0.5 mV·s^−1^ after 200 cycles; and (**b**) comparison of capacitance values with cycle number for GSs, PAQS/GSs(m) and PAQS/GSs in 0.5 M H_2_SO_4_.

Cyclic voltammetry (CV) curves after 200 cycles for GSs, PAQS/GSs(m) and PAQS/GSs at a scan rate of 0.5 mV·s^−1^ between −0.2 and 0.8 V (*vs*. Ag/AgCl), which is the electrochemically stable potential window in 0.5 M H_2_SO_4_ electrolyte, are shown in Figure 6a. The CV curve for GSs shows a typical rectangular-like voltammogram without obvious redox peaks, indicating that the electric double-layer capacitance contributes dominantly to the capacitance of GSs. For PAQS/GSs(m), a pair of weak peaks was observed in the range of 0 to −0.2 V (*vs*. Ag/AgCl), implying that PAQS has redox activity in aqueous electrolyte. PAQS/GSs showed a pair of strong redox peaks at around 0 V (*vs*. Ag/AgCl), which are possibly attributed to the original redox characteristics of PAQS in 0.5 M H_2_SO_4_ electrolyte. The anthraquinonyl groups of PAQS are known to have redox activity through electrochemical association with various cations, such as Li^+^ and Na^+^ [13,34]. Thus, it is suggested that the redox mechanism of PAQS could be similar to that in organic electrolytes. Scheme I shows a possible redox mechanism of PAQS in aqueous electrolytes, and its theoretical capacitance is expected to be 225 mAh (g-PAQS)^−1^. The delocalization of electrons over the entire molecule is assumed to be more favorable in PAQS than that of anthraquinone due to the enlarged π-system, and this could bring out the redox peaks shift to more positive potential [17,42] than that of anthraquinone in aqueous solution (−0.16 V (*vs*. Ag/AgCl) [22]). Therefore, the newly appearing redox peaks at around 0 V (*vs*. Ag/AgCl) in the present study are attributed to the original redox reaction activity of PAQS. Furthermore, it is suggested that the distribution of PAQS is an important factor of the redox reaction in aqueous solution because PAQS that was not fully dispersed showed very low reaction activity.

Figure 6b shows the specific capacitance of GSs, PAQS/GSs(m) and PAQS/GSs calculated from their CV curves and normalized by the electrode weight. GSs and PAQS/GSs(m) showed relatively good cycling stability and exhibited capacitance of 98 and 110 F·g^−1^ (corresponding to 27 and 31 mAh·g^−1^) at the 200th cycle, respectively. In the PAQS/GSs, the specific capacitance increases in the initial stage and then maintains stable capacitance of 250 F·g^−1^ (70 mAh·g^−1^). This type of activation process can be seen in conducting polymer electrodes, a phenomenon reportedly related to the gradual formation of electronic conducting pathways during redox reactions [23,43,44,45]. Thus, it is suggested that the PAQS/GSs electrode is almost activated and reaches the optimum condition.

**Scheme I:**
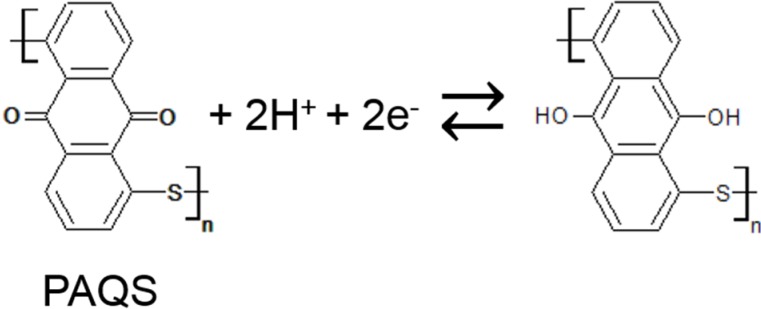
Electrochemical redox mechanism of PAQS in 0.5 M H_2_SO_4_ aqueous solution.

#### 2.6.2. Rate Capabilities

**Figure 7:**
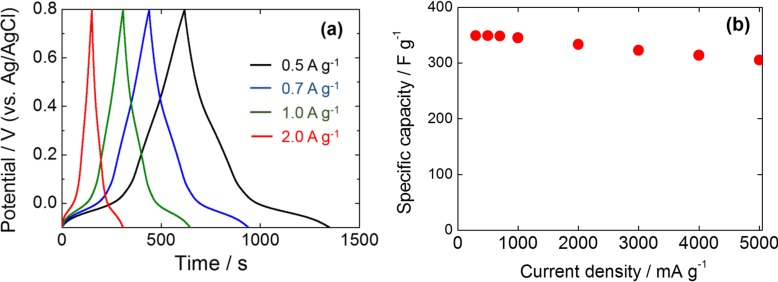
(**a**) Charge/discharge curves at different current densities; and (**b**) plots of specific capacitance *versus* discharge current density for PAQS/GSs.

Figure 7a shows the constant-current charge/discharge curves for PAQS/GSs between −0.1 V and 0.8 V (*vs*. Ag/AgCl) in 0.5 M H_2_SO_4_ at different current densities. The charge/discharge tests were carried out on the fully activated PAQS/GSs electrode. The charge/discharge curves of PAQS/GSs showed a symmetrical shape with a potential plateau corresponding to the reaction of PAQS, and the specific capacitance of PAQS/GSs at a current density of 500 mA·g^−1^ was 349 F·g^−1^ (86 mAh·g^−1^).

From the CV and charge/discharge curves for PAQS/GSs, it is postulated that redox capacitance of PAQS is generated at the potential region lower than 0.2 V (*vs*. Ag/AgCl). The calculated specific capacitance of PAQS corresponds to 156 mAh (g-PAQS)^−1^, which is almost 70% of the theoretical capacitance of PAQS. Charge/discharge curves almost maintain the same shape even at a high current density of 2000 mA·g^−1^. This indicates that PAQS/GSs has electrochemically reversible charge-discharge properties. As shown in Figure 7b, the specific capacitance of PAQS/GSs at a high current density of 5000 mA·g^−1^ is 305 F·g^−1^. This good rate capability of PAQS/GSs seems to have resulted from enhanced electrical conductivity corresponding to the influence of the GSs. Song *et al.* [30] reported that even by mixing a small amount of graphene with polymers, conductivities are greatly improved. This indicates that the electrochemical performance of PAQS/GSs is improved compared with pristine PAQS due to the synergistic effect between GSs and PAQS. The capacitance of PAQS/GSs decreased by only 2% even after 500 charging/discharging cycles at a current density of 500 mA·g^−1^, indicating that PAQS/GSs has long-term electrochemical stability. In the case of PAQS/GSs(m), the capacitance increased by 1% after 500 cycles, which reveals the very slow activation process of PAQS. Based on these results, *in situ* polymerization of PAQS in the presence of GSs is found to be effective for improving the electrode properties for use in electrochemical capacitors using aqueous electrolyte solutions.

## 3. Experimental Section

### 3.1. Material Synthesis

PAQS was prepared through the polycondensation of 1,5-dichloroanthraquinone (15DCAQ) and anhydrous sodium sulfide (Na_2_S) based on the reported procedure [13]. 15DCAQ and Na_2_S were reacted at 120 °C for 24 h in 1-methyl-2-pyrrolidone (NMP). The product was then filtered and washed with acetone and pure water several times. After drying under vacuum at 120 °C, reddish PAQS powder was obtained. GSs were prepared from graphite through oxidation with KClO_3_ and fuming nitric acid followed by rapid thermal exfoliation at 1050 °C for 15 s in a muffle furnace [46,47]. To prepare the composite of PAQS and GSs (PAQS/GSs), GSs were dispersed in NMP by ultrasonication before adding 15DCAQ and Na_2_S. The influence of *in situ* polymerization was investigated through comparison with the mechanically mixed PAQS and GSs (PAQS/GSs(m)). To prepare PAQS/GSs(m), PAQS and GSs were dispersed in acetone by ultrasonication. The acetone was evaporated and PAQS/GSs(m) was obtained. The weight ratio of PAQS to GSs was fixed at 6:4 for all of the samples in this study.

### 3.2. Characterization

Fourier transform infrared spectroscopy (FT-IR) spectra of the prepared PAQS, GSs and PAQS/GSs were recorded on an IRPrestige-21 spectrometer (Shimadzu Corp., Kyoto, Japan). Raman spectra of the samples were recorded on an NR1800 (Jasco Corp., Tokyo, Japan) using a 514.5 nm argon-ion laser. The crystal structures of PAQS, GSs and PAQS/GSs composite were confirmed by means of X-ray diffraction (XRD) analysis using a Bruker D8 ADVANCE diffractometer (Bruker, Billerica, USA). The Brunauer-Emmett-Teller (BET) surface areas of the samples were measured using a TriStar 3000 (Micromeritics, Norcross, GA, USA). The morphology of GSs, PAQS/GSs(m) and PAQS/GSs was observed by scanning electron microscopy (SEM) on an SU8000 (Hitachi, Tokyo, Japan) and transmission electron microscopy (TEM) on an H-9000NAR (Hitachi, Tokyo, Japan).

### 3.3. Electrochemical Measurement

Electrochemical measurement was performed using a three-electrode cell containing 0.5 M H_2_SO_4_ aqueous solution as the electrolyte. An Ag/AgCl electrode and Pt mesh were used as the reference and the counter electrode, respectively. The working electrode was fabricated by mixing active material (PAQS/GSs, PAQS/GSs(m), GSs), Ketjen black and PTFE (polytetrafluoroethylene) with a weight ratio of 6:3:1 and pressing the mixture onto a Ti mesh under a loading of 5 mg·cm^−2^ at a pressure of ~300 MPa. Cyclic voltammetry tests and galvanostatic charge/discharge tests were carried out using a VMP3 (BioLogic, Grenoble, France).

## 4. Conclusions

Poly(anthraquinonyl sulfide) (PAQS)/graphene sheets (GSs) composite was developed as an electrode material for electrochemical capacitors using 0.5 M H_2_SO_4_ aqueous solution. PAQS was uniformly dispersed on the surface of GSs through simple *in situ* polymerization. PAQS/GSs showed a pair of strong redox peaks at around 0 V and good cycling stability. The specific capacitance of PAQS/GSs was 349 F·g^−1^ at a current density of 500 mA·g^−1^, and a capacitance of 305 F·g^−1^ was maintained even at a high current density of 5000 mA·g^−1^. The superior electrode properties of PAQS/GSs are attributed to the fast charge transfer and redox reaction resulting from the introduction of GSs in the composite.
